# Advancing accuracy in breath testing for lung cancer: strategies for improving diagnostic precision in imbalanced data

**DOI:** 10.1186/s12931-024-02668-7

**Published:** 2024-01-16

**Authors:** Ke-Cheng Chen, Shuenn-Wen Kuo, Ruei-Hao Shie, Hsiao-Yu Yang

**Affiliations:** 1 Division of Thoracic Surgery, Department of Surgery, National Taiwan University Hospital, Taipei, Taiwan; 2 National Taiwan University College of Medicine, Taipei, Taiwan; 3 Green Energy and Environmental Research Laboratories, Industrial Technology Research Institute, Hsinchu, Taiwan; 4 Institute of Environmental and Occupational Health Sciences, National Taiwan University College of Public Health, No. 17 Xuzhou Road, Taipei, 10055 Taiwan; 5 Department of Public Health, National Taiwan University College of Public Health, Taipei, Taiwan; 6 Population Health Research Center, National Taiwan University, Taipei, Taiwan; 7 Department of Environmental and Occupational Medicine, National Taiwan University Hospital, Taipei, Taiwan

**Keywords:** Volatile metabolite, Breathomics, Imbalanced learning, Electronic nose

## Abstract

**Background:**

Breath testing using an electronic nose has been recognized as a promising new technique for the early detection of lung cancer. Imbalanced data are commonly observed in electronic nose studies, but methods to address them are rarely reported.

**Objective:**

The objectives of this study were to assess the accuracy of electronic nose screening for lung cancer with imbalanced learning and to select the best mechanical learning algorithm.

**Methods:**

We conducted a case‒control study that included patients with lung cancer and healthy controls and analyzed metabolites in exhaled breath using a carbon nanotube sensor array. The study used five machine learning algorithms to build predictive models and a synthetic minority oversampling technique to address imbalanced data. The diagnostic accuracy of lung cancer was assessed using pathology reports as the gold standard.

**Results:**

We enrolled 190 subjects between 2020 and 2023. A total of 155 subjects were used in the final analysis, which included 111 lung cancer patients and 44 healthy controls. We randomly divided samples into one training set, one internal validation set, and one external validation set. In the external validation set, the summary sensitivity was 0.88 (95% CI 0.84–0.91), the summary specificity was 1.00 (95% CI 0.85–1.00), the AUC was 0.96 (95% CI 0.94–0.98), the pAUC was 0.92 (95% CI 0.89–0.96), and the DOR was 207.62 (95% CI 24.62–924.64).

**Conclusion:**

Electronic nose screening for lung cancer is highly accurate. The support vector machine algorithm is more suitable for analyzing chemical sensor data from electronic noses.

**Supplementary Information:**

The online version contains supplementary material available at 10.1186/s12931-024-02668-7.

## Introduction

Lung cancer is the leading cause of cancer-related deaths globally, with 2.2 million new cases and 1.8 million deaths from lung cancer reported in 2020 [1]. Large numbers of lung cancer screening tools are being developed, but effective screening methods are still lacking. Chest radiography screening has now been found not to reduce lung cancer mortality [2]. Low-dose CT-based lung cancer screening reduces lung cancer mortality; however, the adoption of lung cancer screening programs has been slow [1]. A need exists to develop new lung cancer screening tools.

Breath tests are an innovative tool for lung cancer screening. Because lung cancer patients can exhale specific volatile organic compounds [3–5], studies have used gas sensing array technology (also known as an electric nose) to analyze the exhaled gases of lung cancer patients [6, 7]. The electronic nose uses an array of sensors to measure volatile metabolites in exhaled breath, offering the advantages of a short analysis time, low cost and ease of operation [8]. However, imbalanced data are commonly observed in electronic nose studies, but methods to address imbalanced data have seldom been reported [35]. Imbalanced data can negatively affect the accuracy of a diagnostic test by leading to biased models that perform poorly on minority classes [9]. When a diagnostic test is trained on an imbalanced dataset, the model may overfit to the majority class and underfit to the minority class, leading to better performance on the former. As a method to address this issue, we can balance the dataset by oversampling the minority population or undersampling the majority population to improve the performance of the model on the minority group [10]. Balancing the dataset can help improve performance; however, the accuracy must be corrected when sensitivity or specificity is exceptionally high to generalize the data in community-based screening [11].

The objectives of this study were to assess the accuracy of electronic nose screening for lung cancer with imbalanced learning and to select the best mechanical learning algorithm.

## Methods

This study followed the STARD guidelines for reporting diagnostic accuracy [12].

### Participants

We conducted a case‒control study between October 2020 and July 2023, recruiting lung cancer patients from the outpatient department of National Taiwan University Hospital. The case group comprised those patients who underwent surgery and were diagnosed with lung cancer based on a pathological report. Lung cancer staging is based on the American Joint Committee on Cancer’s TNM classification (7th edition) [13]. We recruited a healthy control group of health care workers who underwent health screening and chest X-rays in the same hospital during the same period and reported no lung cancer.

### Exclusion criteria

At the screening stage, we excluded pregnant women and people younger than 20 years of age. At the final data analysis stage, we excluded patients with the following characteristics: 1. pathology reported as nonmalignant (e.g., hyperplasia, thymoma); 2. metastatic cancers; and 3. carcinoma in situ or minimally invasive adenocarcinoma.

### Test methods

#### Collection of breath samples

**Fig. 1:**
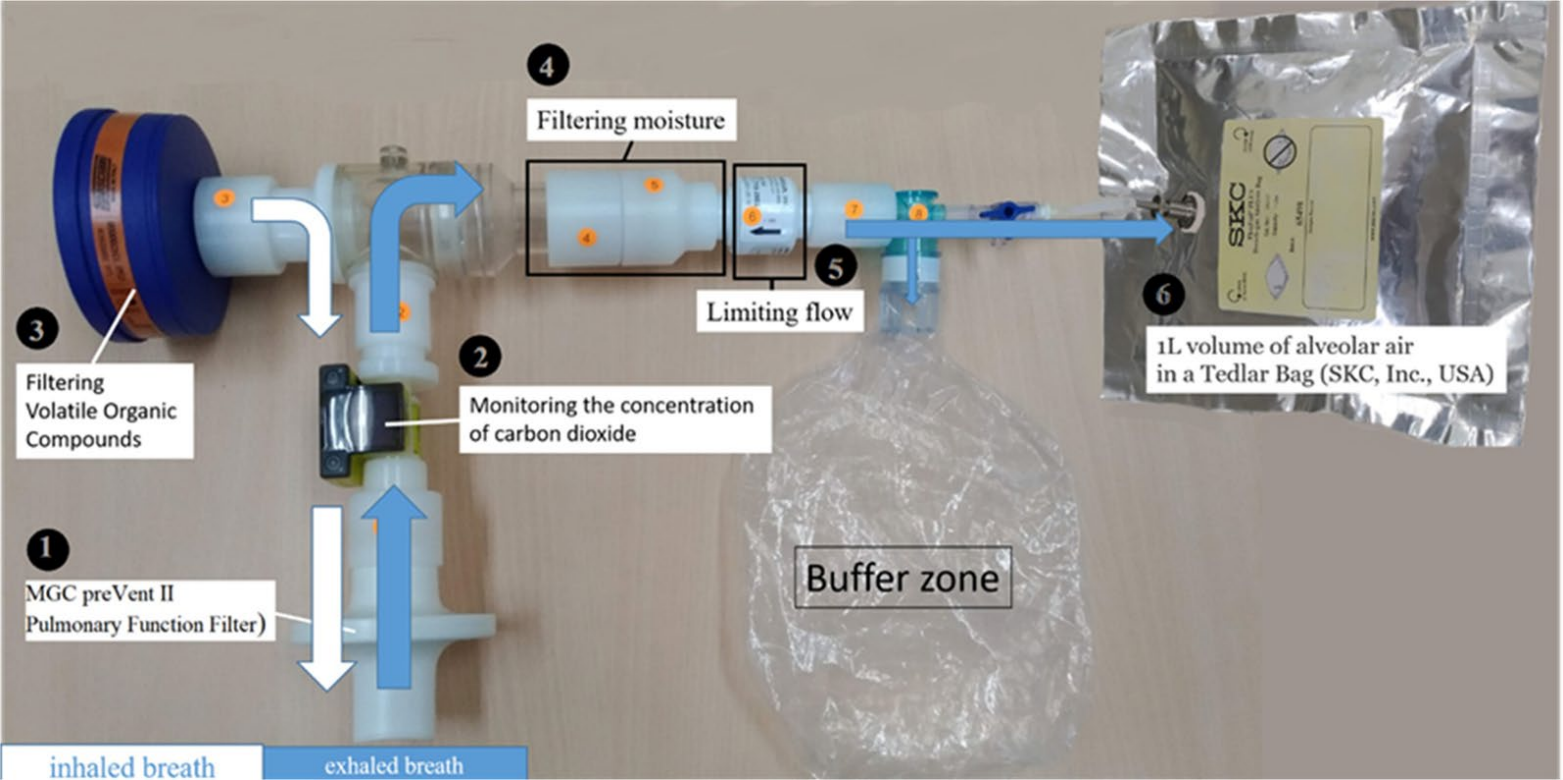
Schematic of the system framework and sample collection. We sampled alveolar air from the collecting device with a volatile organic compound filter, capnometer, flow meter, nonrebreathing bag, and a three-way control valve. The capnometer (Masimo, CA, USA) was monitored [49]. Breath samples were stored in a 1-L Tedlar bag (SKC Inc., PA, USA)

We collected alveolar air samples using a standardized procedure [14]. As concentrations of volatile metabolites could be influenced by the flow rate, diet, and anatomical dead space [15, 16], we sampled alveolar air using specially designed equipment (Fig. 1). When expiratory carbon dioxide concentrations reached a high level representative of the expiratory alveolar phase, we collected alveolar air to prevent contamination of the respiratory or digestive dead space [17]. All subjects were not allowed to eat or smoke for 12 h prior to sampling. We used a fixed flow rate to obtain stable volatile metabolite concentrations and prevent the influence of the flow rate [16]. We followed a standardized cleaning protocol according to recommendations from the European Respiratory Society [18]. Each bag (SKC, Inc., USA) was flushed with nitrogen five times and then heated to 45 °C for approximately 12 h to prevent the influence of contaminated sampling bags.

#### Measurement

We collected alveolar air and analyzed samples within two hours*.* We used an electronic nose chemosensor Cyranose320 (Sensigent, California, USA) consisting of 32 nanocomposite conductive polymer sensors to analyze the breath samples. The breath sample in each bag was analyzed 10 times. The flow rate of the electronic nose was set to 120 ml/min, with a baseline purge for 10 s and a sample purge for 40 s, followed by a 10 s wash-out. Cyranose320 uses a 32 conductive polymer chemo-resistive sensor that is sensitive to temperature and humidity [19]. We operated the electronic nose in the same room, which was maintained at a stable temperature (mean 22.6 °C, standard deviation 2.4 °C) and ambient humidity (55%, standard deviation 7%).

#### Preprocessing sensor data

The sensor resistance resulting from pumping indoor air into the electronic nose was used as the baseline response to generate sensor response data. We normalized and autoscaled the raw sensor response data to remove background noise and exclude outliers [20, 21]. Then, the data were autoscaled to the unit variance that refers to mean centering and then divided by the standard deviation. After autoscaling, the value distribution of each sensor across the database was set to have a mean value of zero and one unit standard deviation [20]. We carefully inspected the raw sensor responses and removed sensor data showing S5 and S31 that showed data drift. Then, we obtained a mean value for each sensor response [22].

### Statistics

The data were randomly divided into a training set used to derive the model (3/5 of the observations), an internal validation set (1/5 of the observations), and an external validation set (1/5 of the observations). The data used for validation were never used in model training. We prevented the effects of unequal case proportions in each group by using a synthetic minority oversampling technique (SMOTE) to balance the data by replicating minority observations [23]. We used five algorithms to compare which algorithm was suitable for electronic nose data and provided average values to allow readers to judge the potential range of accuracy when applied to screening. The five machine learning algorithms used to build predictive models included k-nearest neighbors, decision trees, neural networks, support vector machines (SVMs) (including linear kernels, polynomial kernels, and radial basis kernels), and random forests. [24]. We used the R packages “class” to model k-nearest neighbors, “C50” to model decision trees, “neuralnet” to model neural networks, “kernlab” to model SVMs, and “randomForest” to model random forest (RF). We used the modelLookup function of the caret package for automatic parameter tuning to improve model performance [25]. Using bootstrapping, we calculated the accuracy over 100 iterations to determine the parameters of the machine learning method with the highest prediction accuracy. The optimized model was then further tested using an internal validation set and an external validation set to assess its accuracy.

Using the pathology report as a reference standard, we determined the validity of the breath test by calculating accuracy, sensitivity, specificity, positive predictive value (PPV), negative predictive value (NPV), and area under the receiver operating curve (AUC). AUC values of 0.7–0.8, 0.8–0.9, and 0.9–1.0 represent good, very good, and excellent diagnostic accuracy for lung cancer, respectively. [26]. We used 2000 bootstrap replicates to calculate confidence intervals for the AUC [27]. Based on the case‒control study design, the proportion of patients in our study population will be higher than the proportion of lung cancer patients in the community. Partial AUC (pAUC) consists of analyzing only a region of special interest in the ROC curve and allows the selection of models with high specificity or sensitivity, rather than models with better than average performance but a potentially lower clinical value [28]. We calculated the partial AUC for 90–100 specificities and 90–100% sensitivity to estimate the accuracy of breath test application in community screening [27, 29].

We calculated the diagnostic odds ratio (DOR) to compare the accuracy of different machine learning algorithms. A DOR value ranges from 0 to infinity, with higher values indicating better discriminatory test performance [30]. A test with a DOR of 10 is considered an excellent test [31]. We conducted a meta-analysis to derive summary estimates of DOR, sensitivity, and specificity for different machine learning algorithms. We plotted summary receiver operating characteristic (SROC) curves to generate a point estimate of overall accuracy and compared the overall accuracy between the internal validation and external validation sets [32].

We used the kappa statistic to assess reliability. Kappa indicates the degree to which the observed consistency exceeds the expected level of chance; kappa greater than 0.75 indicates excellent consistency beyond chance, kappa between 0.40 and 0.75 indicates moderate to good consistency, and kappa less than 0.40 indicates poor consistency [33].

#### Sensitivity analysis

Because smoking may affect exhaled volatile organic compounds [34, 35], we performed a sensitivity analysis that excluded current smokers.

#### Sample size estimation

We calculated the needed sample size using the following formula [36]:

1 $$\mathrm{SE }= \sqrt{\frac{C(100-C)}{n}}$$

where SE is the standard error, *C* is the percentage of correctly classified patients, and *n* is the estimated sample size. An SE of 3 was used to limit the standard error to no more than 3%, and the acceptable accuracy (*C*) was 90 based on our goals. The number of needed samples for the training set is 100 [34, 35].

## Results

**Fig. 2:**
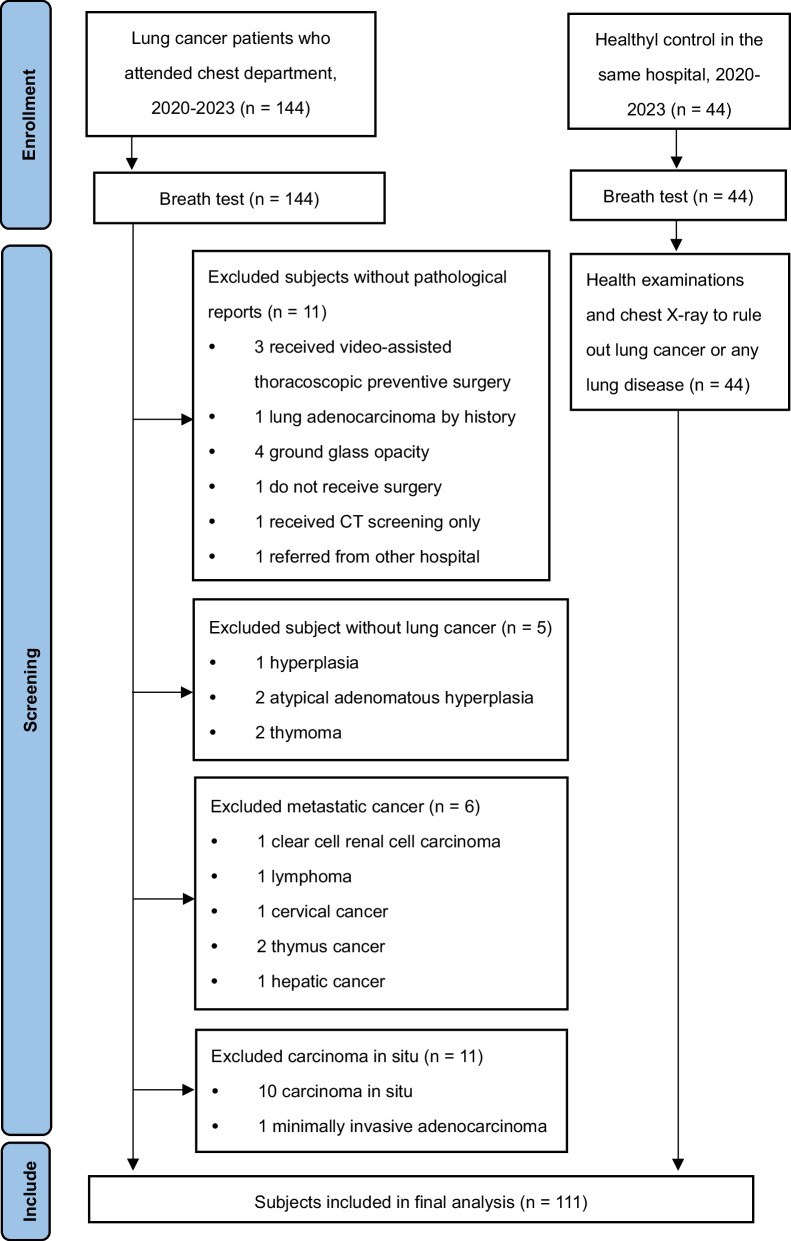
Flow chart of the screening process

We recruited 190 subjects between October 2020 and July 2023. We excluded 2 subjects who were unable to perform the breath test, 11 subjects without pathology reports, 5 subjects with pathology reports that were not diagnosed with lung cancer, 6 subjects with metastatic cancers, and 11 subjects with carcinoma in situ. Finally, 155 subjects were included in the final analysis, including 111 lung cancer patients and 44 healthy controls (Fig. 2). Table 1 shows the demographic characteristics of the study subjects, as well as the staging and histologic types of lung cancer in patients. Most of the lung cancers were stage I adenocarcinoma. The summary sensitivities for the internal and external validation sets were 0.86 (95% CI 0.82–0.89) and 0.88 (95% CI 0.84–0.91), respectively. Sensitivity ranged from 0.77 to 0.92, specificity ranged from 0.58 to 1.00, PPV ranged from 0.66 to 1.00, NPV ranged from 0.83 to 0.92, kappa ranged from 0.47 to 0.90, and AUC ranged from 0.91 to 0.94 (Table 2). The summary sensitivities for the internal and external validation sets were 0.86 (95% CI 0.82–0.89) and 0.88 (95% CI 0.84–0.91), respectively. The summary specificities for the internal and external validation sets were 0.99 (95% CI 0.81–1.00) and 1.00 (95% CI 0.85–1.00), respectively. The summary DORs for the internal and external validation sets were 110.97 (95% CI 31.77–387.62) and 206.95 (95% CI 51.84–826.19), respectively.

**Table 1:** Characteristics of the study subjects

Characteristics	Lung cancer Patients (*n* = 111)	Healthy controls (*n* = 44)
Sex (female) (%)	72 (64.86)	20 (45.45)
Age (years), mean (SD)	64.41 (11.85)	33.23 (8.35)
Never smoked (%)	83 (74.77)	42 (95.45)
Habitual cooking (%)	52 (47.27)	6 (13.64)
Burning incense (%)	37 (33.64)	0 (0)
Burning essential oils (%)	6 (5.45)	1 (2.27)
Family history of lung cancer (%)	30 (27.52)	7 (15.91)
Stage		
I (%)	87 (78.38)	–
II (%)	6 (5.41)	–
III (%)	10 (9.01)	–
IV (%)	8 (7.21)	–
Pathology		
Adenocarcinoma (%)	108 (97.30)	N/A
Squamous cell carcinoma (%)	1 (0.90)	N/A
Adenoid cystic carcinoma (%)	1 (0.90)	
Lymphoepithelial-like carcinoma (%)	1 (0.90)

**Table 2:** Prediction accuracy of the electronic nose data using machine learning algorithms

Model and parameters	Validation	Accuracy (95% CI)	Sensitivity	Specificity	PPV	NPV	Kappa	AUC (95% CI)
*k*-nearest neighbors (*k* = 5)	*Internal*	0.87 (0.79–0.93)	0.88	0.87	0.86	0.88	0.74	0.94 (0.89–0.99)
	*External*	0.94 (0.87–0.98)	0.88	1.00	1.00	0.90	0.88	0.95 (0.90–1.00)
Decision tree (trials = 20, winnow = TRU, model = tree)	*Internal*	0.73 (0.63–0.82)	0.90	0.58	0.66	0.86	0.47	0.91 (0.84–0.98)
	*External*	0.91 (0.84–0.96)	0.92	0.90	0.90	0.92	0.82	0.94 (0.90–1.00)
Neural network (size = 1, decay = 0)	*Internal*	0.89 (0.81–0.94)	0.77	1.00	1.00	0.83	0.78	0.92 (0.86–0.97)
	*External*	0.94 (0.87–0.98)	0.88	1.00	1.00	0.90	0.88	0.94 (0.89–0.99)
Support vector machines (linear kernel) (C = 1)	*Internal*	0.92 (0.85–0.96)	0.83	1.00	1.00	0.87	0.84	0.94 (0.89–0.99)
	*External*	0.92 (0.85–0.96)	0.83	1.00	1.00	0.87	0.84	0.94 (0.89–0.99)
Support vector machines (radial kernel) (C = 1)	*Internal*	0.95 (0.89–0.98)	0.90	1.00	1.00	0.91	0.90	0.94 (0.89–0.99)
	*External*	0.95 (0.89–0.98)	0.90	1.00	1.00	0.91	0.90	0.94 (0.89–0.99)
Support vector machines (polynomial kernel) (degree = 3, scale = 0.1, C = 1)	*Internal*	0.92 (0.85–0.96)	0.83	1.00	1.00	0.87	0.84	0.94 (0.89–0.99)
	*External*	0.94 (0.87–0.98)	0.88	1.00	1.00	0.90	0.88	0.94 (0.89–0.99)
Random forest (mtry = 2)	*Internal*	0.91 (0.84–0.96)	0.90	0.92	0.91	0.91	0.82	0.92 (0.86–0.99)
	*External*	0.79 (0.70–0.87)	0.85	0.73	0.75	0.84	0.58	0.94 (0.90–0.99)

*PPV* positive predictive value, *NPV* negative predictive value, *AUC* area under the receiver operating curve

**Fig. 3:**
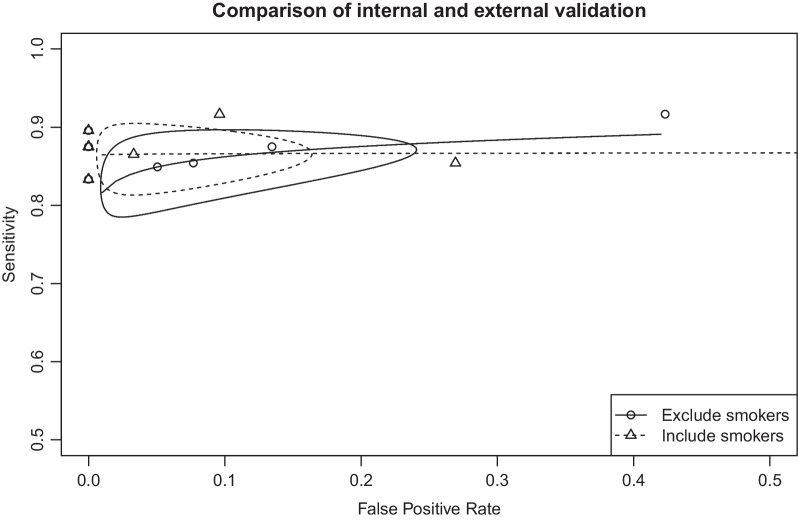
Summary receiver operating characteristic (SROC) curve showing the diagnostic test accuracy for excluding smokers and including smokers. This figure shows summary receiver operating characteristic (SROC) curves for excluding smokers and including smokers. Scatter points are the accuracy obtained from different machine learning models. The ellipsoid confidence region depicts the 95% confidence region in the SROC space for the summary point estimate of diagnostic performance

SROC curves showed higher overall sensitivity for the external validation set but also higher false-positive rates for the external validation set (Fig. 3). The area under the curve (AUC) for both the internal and external validation sets showed an increase in variability when assay specificity exceeded 90% for both the internal (Additional file 1: Figure S1) and external validation sets (Additional file 2: Figure S2). The partial AUCs between 90 and 100% specificity ranged from 0.74 to 0.83 in the internal validation set (Additional file 3: Figure S3) and from 0.83 to 0.86 in the external validation set (Additional file 4: Figure S4).

**Fig. 4:**
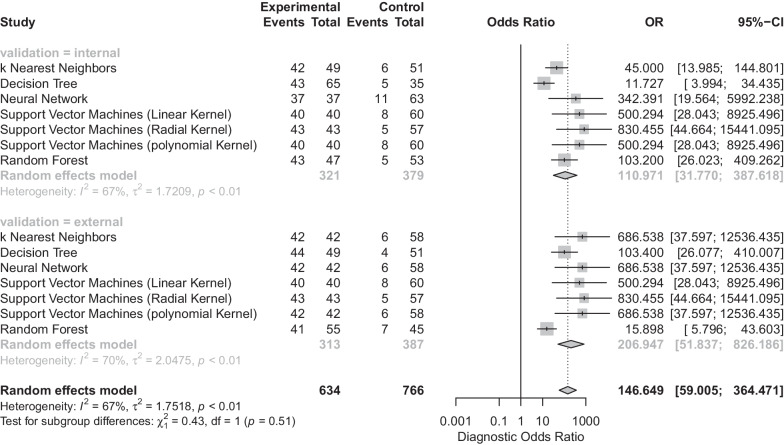
Forest plot of diagnostic odds ratios (DORs) for different machine learning algorithms and validation sets. The vertical dotted line represents the odds ratio (OR) and 95% confidence interval (95% CI). The diamond represents the point estimate and confidence intervals when combining and averaging all the machine learning algorithms together

The summary DOR for the breath tests was 146.65 (95% CI 59.01–364.47) (Fig. 4), and the DORs for the internal validation and external validation sets were 110.97 (95% CI 31.77–387.62) and 206.95 (95% CI 51.84–826.19), respectively. The support vector machine using radial kernel had the highest DOR (Fig. 3) and the highest kappa value.

In the sensitivity analysis, although smoking is likely to influence the volatile organic compounds in breath, this study used a carbon black polymer sensor does not seem to be significantly influenced by smoking (Fig. 3).

## Discussion

### Principal findings

Although electronic noses have been used in diagnostic lung cancer studies, most of these studies have utilized a case‒control study design with a high prevalence of lung cancer in the study population [37, 38], leading to a potential overestimation of diagnostic accuracy. The choice of study design (e.g., case‒control studies conducted in hospitals or cross-sectional studies conducted in the community) can affect the assessment of accuracy, rendering the results not directly applicable, but few reports have been published in the field of machine learning. This study describes a method to correct the high accuracy obtained in a case‒control study to estimate the real accuracy in a community setting. This study shows how the SMOTE approach can be applied to prevent a high proportion of cases or a scarcity of cases in a study population from generating spuriously high sensitivity and specificity. Considering the partial AUC, the electronic nose has good accuracy and reliability. To the best of our limited knowledge, this study is the first to provide epidemiologists’ perspectives and suggest solutions for scientists from different disciplines. We provide evidence that imbalanced learning can enhance the accuracy of lung cancer screening. Compared to our previous study [39], in which air samples were collected from a tracheal tube placed in anesthetized patients, the AUCs were 0.91 (95% CI = 0.79–1.00) using linear discriminant analysis and 0.90 (95% CI = 0.80–0.99) using the supportive vector machine technique. This study further shows that the sensor array technique combined with machine learning can detect lung cancer in an outpatient setting.

The diagnostic accuracy of a test can be affected by the prevalence of the disease in the screened population [40]. Compared to other similar studies, sensitivity and specificity appear to be related to the proportion of lung cancer patients and nonlung cancer controls. When the proportion of controls is high, the specificity is higher than the sensitivity. When a higher proportion of controls is included, the specificity will be higher than the sensitivity. For example, Rocco et al. included 23 lung cancer patients and 77 nonlung cancer controls and reported a sensitivity and specificity of 86% and 95%, respectively [4]. Gasparri’s study involved 70 lung cancer patients and 76 controls, with a sensitivity of 81% and specificity of 91% [5]. Shlomi et al. included 16 lung cancer patients and 30 controls, with a sensitivity of 75% and a specificity of 93% [41]. This difference is primarily due to the way sensitivity and specificity are calculated. Sensitivity is the ability of a test to correctly identify individuals with the disease (true positives). In a population with a higher disease prevalence, more true positive cases are present to detect. In a population with a 100% disease prevalence, even a completely false test that always predicts “having the disease” (regardless of the true disease status) would achieve 100% sensitivity. This outcome is because the test would correctly identify all individuals with the disease, but it would also incorrectly identify all individuals without the disease as having it. As a result, the sensitivity of this test would be 100%, but its specificity, PPV, and NPVV would all be 0%. On the other hand, in a population with a 0% disease prevalence, even a completely false test that always predicts “no disease” (regardless of the true disease status) would achieve 100% specificity. However, its sensitivity, positive PPV, and NPV were all 0%. In other words, while it would be good at correctly identifying individuals without the disease (specificity), it would be entirely useless for correctly identifying individuals with the disease. This result highlights the importance of complete reporting of sensitivity, specificity, PPV, and NPV when evaluating the performance of diagnostic tests.

The choice of the study design can indeed have a significant influence on the detection accuracy of a diagnostic test. A case‒control study design conducted in a hospital setting with a high prevalence of the disease in the study population can result in a higher sensitivity for the test. This outcome is because the likelihood of encountering individuals with the disease (cases) is higher in such a setting, which can make it easier for the test to detect the condition, thereby leading to a higher sensitivity. Therefore, while a hospital-based case‒control study with a high disease prevalence can increase sensitivity, it can also result in a potential drawback known as “spectrum bias” [42]. Spectrum bias occurs when the study population does not accurately represent the broader population in which the test will eventually be used. A hospital-based case‒control study with a high disease prevalence can enhance sensitivity but might not fully represent the test’s performance in community-based scenarios with varying disease prevalences. The pAUC can be used as a method to assess the generalizability of the results to a broader population by providing a corrected AUC when sensitivity or specificity is exceptionally high due to imbalanced data, such as in situations where the disease prevalence is extremely high or low in the study population. The pAUC is a valuable tool in cases where traditional AUC measures may not adequately account for the impact of imbalanced data on evaluations of test performance.

SVM has the highest accuracy for analyzing data from electronic nose sensor arrays. Uddin et al. compared different machine learning algorithms used for disease prediction and found that SVM was most frequently used, but the RF algorithm was relatively accurate, followed by SVM. However, Uddin’s review included algorithms used to analyze clinical and demographic data and did not specifically consider the electronic nose data, which may have different characteristics and requirements for analysis [43]. In our study, we evaluated the accuracy using DOR. The DOR is commonly used in meta-analyses of diagnostic studies that can combine results from different studies into summary estimates with increased precision [30]. The results show that SVM has the highest accuracy and is suitable for analyzing normalized and centralized electronic nose sensor array data. This finding is consistent with a comparison of machine learning algorithms for the classification of electronic nose data [44–46]. The reasons why SVM has the highest accuracy and is suitable for this type of analysis include (1) Non-Linearity Handling: SVM is capable of handling nonlinear data by mapping it into a higher-dimensional space using a kernel trick. This ability is valuable because electronic nose sensor array data can be complex and nonlinear [47]. (2) Strong Margin Maximization: SVM aims to find a decision boundary that maximizes the margin between different classes [48]. This approach often results in a more robust and accurate classification, especially when the data are well structured, as in normalized and centralized sensor array data. (3) Effective with High-Dimensional Data: SVM is particularly effective when analyzing high-dimensional data, which is often the case in electronic nose sensor array data. It helps find patterns and relationships in these complex datasets. However, importantly, the SVM performance depends on factors such as kernel selection, parameter tuning, and the specific characteristics of the dataset. While SVM can be a powerful tool, other algorithms must be considered and thorough experiments must be conducted to determine the best approach. However, SVM is sensitive to noisy data and outliers that can significantly affect the overall performance of the model. When analyzing electronic nose data, noisy data can come from (1) changes in environmental conditions such as temperature and humidity and (2) sampling variability, such as the way samples are prepared or presented to the electronic nose, which may contribute to noise. Inconsistent sample handling may lead to inconsistent sensor responses. (3) Background Odors: The presence of background odors or contaminants in the measurement environment can interfere with the detection of specific target odors, adding noise to the data. (4) Signal Processing Artifacts: The methods used for data preprocessing can introduce noise if not applied appropriately. Before applying SVM, we recommend that researchers develop standardized procedures for performing the electronic nose analysis in an air-conditioned room with a fixed temperature and humidity. Researchers can use a volatile organic compound filter at the inlet of the sampling device to eliminate background odors (Fig. 1, Part 3). A dehumidification chamber containing silica gel can be added to the inlet of the air samples (Fig. 1, Part 4). When preprocessing sensor data, normalization and autoscalation are important to remove background noise and exclude outliers. Using standardized procedures, the findings can be generalized to other populations.

## Conclusions

In this study, we developed a breath test for lung cancer using chemical sensors and machine learning techniques. Since the study was conducted in a hospital with a case‒control study design, we described methods to estimate the accuracy of the breath test in community screening and how to apply the SMOTE approach to imbalanced data. Through a meta-analysis and the DOR, this study shows that the SVM algorithm is more suitable for classifying chemosensor data from electronic noses.

### Supplementary Information

**Additional file 1: Figure S1.** Area under the curve (AUC) for the internal validation set. (Conti.) (a) k-nearest neighbours, (b) Decision tree, (c) Neural network, (d) Support vector machines, linear kernel, (e) Support vector machines, radial kernel, (f) Support vector machines, polynomial kernel, (g) Random forest.

**Additional file 2: Figure S2.** Area under the curve (AUC) for the external validation set. (a) k-nearest neighbors, (b) Decision tree, (c) Neural network, (d) Support vector machines, linear kernel, (e) Support vector machines, radial kernel, (f) Support vector machines, polynominal kernel, (g) Random forest.

**Additional file 3. Figure S3.**

**Additional file 4. Figure S4.**

## Data Availability

Data are available upon request due to privacy/ethical restrictions.

## Abbreviations

PPV: Positive predictive value
NPV: Negative predictive value
AUC: Area under the receiver operating curve
pAUC: Partial AUC
DOR: Diagnostic odds ratio
SROC: Summary receiver operating characteristic curve
SMOTE: Synthetic minority oversampling technique
SVM: Support vector machine
RF: Random forest
