# Supplementary material for: Advancing accuracy in breath testing for lung cancer: strategies for improving diagnostic precision in imbalanced data
Source: Respir Res. 2024 Jan 16;25:32. doi: 10.1186/s12931-024-02668-7 (PMC10790556; doi:10.1186/s12931-024-02668-7)

**Title:** Advancing Accuracy in Breath Testing for Lung Cancer: Strategies for Improving Diagnostic Precision in Imbalanced Data

**Authors:** Ke-Cheng Chen^1,2^; Shuenn-Wen Kuo^1,2^; Ruei-Hao Shie^3^; Hsiao-Yu Yang^4,5,6,7^*

**Affiliation:**

^1^ Division of Thoracic Surgery, Department of Surgery, National Taiwan University Hospital, Taipei, Taiwan; cskchen@gmail.com

^2^ National Taiwan University College of Medicine, Taipei, Taiwan

^3^ Green Energy & Environmental Research Laboratories, Industrial Technology Research Institute, Hsinchu, Taiwan; rueihaoshie@itri.org.tw

^4^ Institute of Environmental and Occupational Health Sciences, National Taiwan University College of Public Health, Taipei, Taiwan; hyang@ntu.edu.tw

^5^ Department of Public Health, National Taiwan University College of Public Health, Taipei, Taiwan

^6^ Innovation and Policy Center for Population Health and Sustainable Environment (Population Health Research Center, PHRC), National Taiwan University

^7^ Department of Environmental and Occupational Medicine, National Taiwan University Hospital, Taipei, Taiwan

- Correspondence: Hsiao-Yu Yang, MD, PhD

E-mail: [hyang@ntu.edu.tw](mailto:hyang@ntu.edu.tw)

Address: No. 17 Xuzhou Road, Taipei 10055, Taiwan


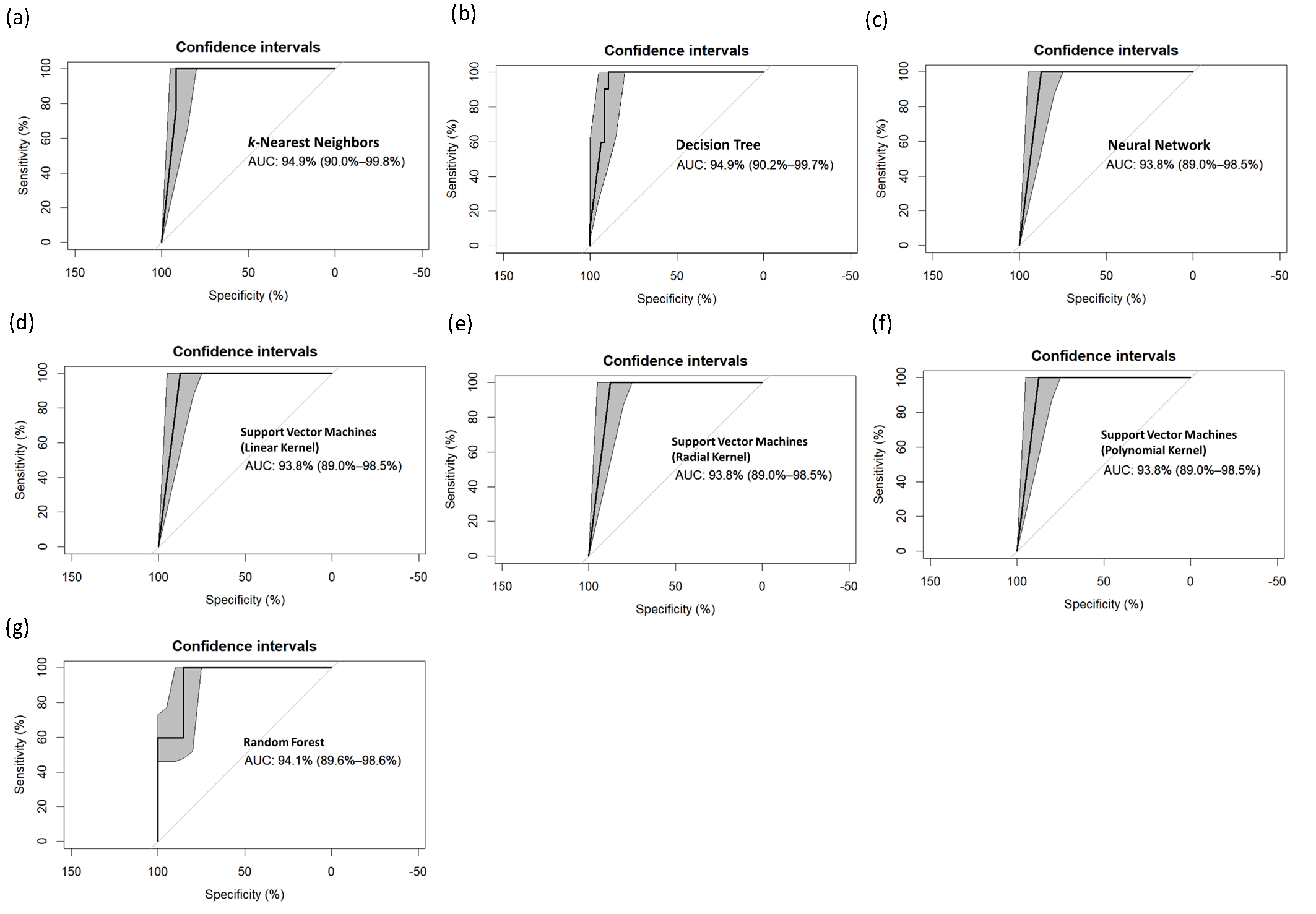

Supplement: Supplementary file 2 — Additional file 2: Figure S2. Area under the curve (AUC) for the external validation set. (a) k-nearest neighbors, (b) Decision tree, (c) Neural network, (d) Support vector machines, linear kernel, (e) Support vector machines, radial kernel, (f) Support vector machines, polynominal kernel, (g) Random forest. [file 12931_2024_2668_MOESM2_ESM.docx]
